# Associations between physical activity patterns and quality of life in persons with type 2 diabetes: A cross sectional study

**DOI:** 10.1371/journal.pone.0290825

**Published:** 2023-08-30

**Authors:** Ulric Sena Abonie, Ama Kissiwaa Ofori ‐ Ampomah, Vincent Makinyi, Raphael Aseye Addo, Laureen Kumah

**Affiliations:** 1 Department of Sport, Exercise & Rehabilitation, Northumbria University, Coach Lane Campus, Benton, Newcastle upon Tyne, United Kingdom; 2 Department of Physiotherapy and Rehabilitation Sciences, University of Health and Allied Sciences, Ho, Volta Region, Ghana; Medical Research Council / Uganda Virus Research Institute & London School of Hygiene and Tropical Medicine Uganda Research Unit, UGANDA

## Abstract

**Background:**

Type 2 diabetes is a major health problem globally and particularly in Ghana. Regular physical activity is important in the management of type 2 diabetes and in improving quality of life of persons with type 2 diabetes. However, there is a lack of data reporting on how physical activity relate to quality of life in persons with diabetes in Ghana. This study explored how physical activity patterns relate to quality of life in persons with type 2 diabetes from a major tertiary hospital in Ghana.

**Methods:**

One hundred and twenty-one (121) persons with type 2 diabetes (age, 30–60 years) filled in questionnaires on their physical activity patterns (time spent in sitting, walking, moderate-intensity activities, and vigorous-intensity activities) and quality of life (diabetes control, anxiety and worry, social burden, sexual functioning, energy and mobility). The relationships between the variables were examined using spearman correlation.

**Results:**

Time spent in sitting, walking, moderate-intensity activities and vigorous-intensity activities were 1677.7±401.5min, 464.1±296.0MET-min, 241.2±65.8MET-min and 1956.5±1251.0MET-min respectively. Walking was negatively related to energy and mobility (r = -.48, p<0.01), sexual functioning (r = -0.44, p<0.01), social burden (r = -0.41, p<0.01) and diabetes control (r = -0.56, p<0.01) domains of quality of life. Vigorous-intensity activities was negatively related to anxiety and worry (r = -0.20, p<0.05).

**Conclusions:**

The results suggests that persons with type 2 diabetes who experience decline in energy and mobility, sexual functioning, and disease management, and heightened social burden, anxiety and worry may benefit from guidance on optimal physical activity behaviour in the form of walking to improve their quality of life.

## Introduction

Type 2 diabetes is a major public health issue globally [1]. In Ghana, the prevalence of type 2 diabetes has been increasing, with an estimated 7% of the adult population affected by type 2 diabetes [2–4]. However, there are even many cases which are undiagnosed due to numerous factors including but not limited to unavailability of resources for detection and diagnosis [4–6]. Equally, type 2 diabetes associated morbidity and mortality has been increasing appreciably in recent years [1, 7–9].

Lower quality of life has been observed in persons with type 2 diabetes compared to persons without diabetes [10]. Reducing the burden of type 2 diabetes is therefore an important public health priority globally and particularly across countries in Africa, the geographical region with the highest type 2 diabetes associated morbidity and mortality [7–9, 11]. Despite ongoing activities to improve management of type 2 diabetes, more needs to be done considering the high and growing burden of the condition [7].

Lifestyle changes are recommended to reduce the burden of type 2 diabetes. Consequently, the adoption and maintenance of physical activity together with decreasing sedentary time is essential for type 2 diabetes management and overall health in persons with diabetes [12, 13]. Physical activity improves blood glucose control and quality of life, reduces cardiovascular risk and mortality in persons with type 2 diabetes, and prevent or delay onset of type 2 diabetes [14–20].

Despite the growing burden of type 2 diabetes and the known beneficial effects of physical activity, studies exploring physical activity behaviour and how it relates to quality of life in persons with type 2 diabetes in Ghana are scarce. Understanding this relation can help guide and tailor management for persons with type 2 diabetes and promote an active lifestyle in this population to reduce their disease burden.

Therefore, the aim of this study was to examine reported time in physical activity forms and how these relate to quality of life in persons with type 2 diabetes attending a major tertiary hospital in Ghana. We hypothesized that time spent in sitting would be associated with a decrease in quality of life, and time spent in walking, moderate-intensity activities and vigorous-intensity activities would be associated with an increase in quality of life.

## Materials and methods

This was a cross-sectional study design where participants reported time spent in sitting, walking, moderate-intensity activities and vigorous-intensity activities, and their quality of life. All study procedures were approved by the Research Ethics Committee of the University of Health and Allied Sciences [reference: UHAS-REC A.9 (104) 20, 21].

Participants were persons with type 2 diabetes recruited from the Diabetes Clinic, Ho Teaching Hospital, located within the Ho Municipality of the Volta Region in Ghana. The Diabetes Clinic provides medical care for people with diabetes in the entire region. Participants were recruited through public advertisement (social media, posters and word of mouth) between May 2021 to July 2021.

Those interested in participation were contacted by the researchers who explained the study rationale, potential benefits, procedures, and answered all questions. Written informed consent was obtained from eligible and willing participants. Criteria for inclusion were people 18 years and older, diagnosed with diabetes for a year or more and ambulatory (with or without assistive device). Participants were excluded from the study if they were not able to complete the questionnaires even with help or had comorbid conditions that may influence understanding or answering of the questionnaires.

Enrolled participants were assessed through standardised measurements obtained from a clinic visit. During the visit, demographic data, including age, sex, educational status, marital status, duration of illness (years since diagnosis) and medical treatment were first collected. Afterwards, participants completed a set of questionnaires: a self-report questionnaire on their physical activity [20] and diabetes-related quality of life [21]. Further details of these measures provided below. To prevent questionnaire order and administration biases, participants completed the questionnaires in random order [22].

An a priori sample size calculation was performed using the Roasoft online sample size calculator (Roasoft Inc, 2004, http://www.raosoft.com/samplesize.html), using the average monthly attendance of persons with diabetes at the study site which was 60 [23]. At 95% confidence level, 5% margin of error, and a response distribution of 50%, an estimated sample size of 92 participants was required.

Physical activity levels were assessed using the short version of the International Physical Activity Questionnaire (IPAQ-SF). The original IPAQ was developed as an instrument for cross national monitoring of physical activity and inactivity across diverse countries and populations [20]. The IPAQ has been shown to be a reliable and valid tool to assess physical activity [20]. The IPAQ-SF contains 7 items and assesses the frequency (days) and duration (minutes and/or hours) of sitting, walking, moderate- and vigorous-intensity activities. Total minutes for each activity was computed by multiplying the frequency by the duration. A Metabolic equivalent (MET)-minute was computed for walking, moderate and vigorous activities by multiplying the MET score by the activity duration (minute). One MET is defined as the resting metabolic rate. The following values were used for scoring: walking × 3.3 METs, moderate physical activity × 4.0 METs, vigorous physical activity × 8.0 METs [20].

Diabetes-related quality of life was assessed using the Diabetes-39 (D-39) Questionnaire. The D-39 is specific to types 2 diabetes and contains five dimensions: energy and mobility (15 items), diabetes control (12 items), anxiety and worry (4 items), social burden (5 items), and sexual functioning (3 items) [21]. The D-39 is a valid discriminative instrument and has been shown to significantly correlate with overall quality of life, pattern of diabetes severity, and comorbidity [21]. The score for each item ranged from 1 (not affected at all) to 7 (extremely affected). The possible score for all dimensions ranges from 39 to 273, with a low score indicating better quality of life [21].

### Data analysis

Data were analysed using IBM Statistical Package for the Social Sciences version 23.0 [24]. Based on descriptive statistic and visual inspection of frequency distributions, data were non-normally distributed. All values were reported using descriptive statistics of means, standard deviations and ranges to summarize characteristics of participants. Associations between time spent in sitting, walking, moderate-intensity activities and vigorous-intensity activities, and quality of life domains were examined using spearman correlations coefficients.

## Results

Response rate was 100%. Descriptive statistics of the study sample and outcome measures are presented in Table 1. Of the 121 participants (age = 30–60 years), majority were female (72%), aged 41–50 years (44.6%), had tertiary education (28.1%) and were married (82.6%). More than half of the respondents were employed (52.9%), had been living with the condition between 5–10 years (56.2%) and were on Metformin (82.6%).

**Table 1:** Demographic and physical characteristics of the study participants (N = 121).

Variable	N (%) or Mean ± SD
Age category	
≤30	14 (11.6)
31–40	22 (18.2)
41–50	54 (44.6)
51–60	31 (25.6)
Gender	
Male	34 (28)
Female	87 (72)
Educational status	
Tertiary	34 (28.1)
Senior secondary	23 (19.0)
Junior secondary	18 (14.9)
Primary	26 (21.5)
No education	20 (16.5)
Marital status	
Single	21 (17.4)
Married	100 (82.6)
Employment status	
Employed	64 (52.9)
Unemployed	57 (47.1)
Diabetes duration	
<5 years	32 (26.5)
5–10 years	68 (56.2)
11–15 years	16 (13.2)
≥16	5 (4.1)
Type of diabetes medication	
Insulin	13 (10.7)
Metformin	100 (82.7)
Others	8 (6.6)
Physical Activity	
Sitting (minutes)	1677.7 ± 401.5
Walking (MET-minutes)	464.1 ± 296.0
Moderate activities (MET-minutes)	241.2 ± 65.8
Vigorous activities (MET-minutes)	1956.5 ± 1251.0
Quality of life	
Energy and Mobility	39.7 ± 22.2
Diabetes Control	32.5 ± 18.5
Anxiety and Worry	10.5 ± 5.8
Social Burden	12.1 ± 9.4
Sexual Function	8.2 ± 6.6

Averagely, the participants spent 1677.7 ± 401.5min in sitting, 464.1 ± 296.0MET-min in walking, 241.2 ± 65.8 MET-min in moderate-intensity activities and 1956.5 ± 1251.0 MET/min in vigorous-intensity activities. Overall quality of life was 194.3 ± 42.9.

### Associations between physical activity and quality of life domains

Results from the associations between physical activity patterns and quality of life domains are presented in Table 2. The results revealed a statistically significant negative moderate associations between walking and energy and mobility (r = -.48, p<0.01), sexual functioning (r = -0.44, p<0.01), social burden (r = -0.41, p<0.01) and diabetes control (r = -0.56, p<0.01). In other words, walking was associated with better energy and mobility, sexual functioning, diabetes control and reduced social burden.

**Table 2:** Spearman correlations of physical activity and quality of life domains.

	Energy and mobility	Diabetes control	Anxiety and worry	Social burden	Sexual functioning
Sitting	-0.05	-0.01	-0.16	0.02	-0.04
Walking	-0.48^*^	-0.56^*^	-0.16	-0.41^*^	-0.44^*^
Moderate activities	-0.12	-0.09	-0.06	-0.15	-0.07
Vigorous activities	-0.14	-0.16	-0.20^*^	-0.10	-0.14

* Correlation Is Significant at p<0.05

A significantly weak negative association was also found between vigorous-intensity activities and anxiety and worry (r = -0.20, p<0.05). In other words, vigorous-intensity activities were associated with less anxiety and worry.

No associations were found between sitting and all quality of life domains (p>0.05), and between moderate activity and all quality of life domains (p>0.05).

## Discussion

This study investigated the associations between reported physical activity patterns (sitting, walking, moderate and vigorous activity) and quality of life domains in persons with type 2 diabetes attending a tertiary hospital in Ghana and found modest negative associations between walking and energy and mobility, sexual functioning, social burden, and diabetes control. In other words, increased self-reported walking was associated with better energy and mobility, sexual functioning, diabetes control and reduced social burden. Furthermore, we found vigorous-intensity activities were negatively associated with anxiety and worry. In other words, vigorous-intensity activities were associated with lower anxiety and worry. The results of this study further underscore the importance of physical activity in the management of type 2 diabetes.

The findings that walking was associated with better energy and mobility, sexual functioning, diabetes control and reduced social burden, and vigorous activities were associated with less anxiety and worry are comparable to the findings of studies investigating whether there were relationships between physical activity, diabetic control and quality in persons with type 2 diabetes [15, 23]. Çolak et al., [15] in their study found that physical activity was associated with better energy and mobility, sexual functioning, diabetes control and reduced social burden and anxiety and worry. Similarly, Osei-Yeboah et al., [23] in their study, reported that physical activity was associated with glycaemic control.

The lack of significant associations between sitting and quality of life found in this study was similar to that found in the study by Çolak et al. [15] investigating the association between physical activity and quality of life in persons with type 2 diabetes but contrary to the study by Daniele et al. [25] that examined associations between physical activity, comorbidity severity, depressive symptoms, and health-related quality of life in persons with type 2 diabetes and found sedentary lifestyle was associated with low quality of life, functional capacity and general health.

The descriptive statistic indicated that the study sample reported poor quality of life, high sitting time, low walking, moderate-intensity and vigorous-intensity activities which were comparable to those reported in similar studies investigating activity patterns and quality of life in persons with type 2 diabetes in Turkey and Brazil [15, 25]. Furthermore, the levels of moderate-intensity activities and vigorous-intensity activities found in this study were less than the recommended minimum for each category (600 MET-min/week for moderate-intensity activities, and 3,000 MET-min/week for vigorous-intensity activities) [26]. Previous works on physical activity pattern among persons with type 2 diabetes in Ghana concluded that the level of physical activity in this population was inadequate [23].

Taken together, the findings of poor overall quality of life, high sitting time and low physical activity level highlights the fact that persons with type 2 diabetes spends considerable amount of time in sedentary and do not engage in adequate amount of physical activity. Time spent in sedentary has been shown to be strongly and adversely associated with poor cardio-metabolic health and poor general health [27]. Given that physical inactivity is detrimental and physical activity plays an important role in the management of type 2 diabetes, reducing sitting time and increasing physical activity is essential in persons with type 2 diabetes. Consequently, there is the need to explore ways to improve physical activity participation and quality of life in this population [28].

Guidance on engagement in optimal physical activity behavior may be beneficial for persons with type 2 diabetes. Conversely, walking, a form of physical activity that is classified as economical and has a low risk [29] may be easier and more realistic and achievable for persons with type 2 diabetes to engage in, compared to vigorous forms of physical activity. The study findings that walking was associated with improved quality of life thus suggest that persons with diabetes may benefit from interventions incorporating walking. This highlights the need for the development and design of goal-directed interventions incorporating walking in relation to what we know from literature to help guide treatment efforts for people with type 2 diabetes.

This study had a number of limitations. Importantly, because a convenience sampling strategy was used, the findings are limited in their generalizability to a more diverse type 2 diabetes population. In addition, the relatively small study sample size limits the evaluation of confounding effects (adjusted estimates), thus limit the ability to draw firm conclusion. It would be useful to replicate these analyses in a larger sample to obtain adjusted estimates while controlling for confounders such as disease duration, age, gender and comorbidity. Furthermore, although self-report measures are more feasible in population studies, they are susceptible to biases as they involve recalling activities (over days, weeks, or months) that could lead to underreporting or overreporting [30], thus the use of self-report measures in this study is a limitation. Using an objective device would allow to examine more macro levels of activity and is warranted in future study. Lastly, the study design employed limits the ability to draw causal inferences.

## Conclusion

In this study we examined associations between self-reported activity patterns and quality of life domains and found that physical activity, particularly walking and vigorous-intensity activities were associated with better quality of life. The result of the study further showed that persons with type 2 diabetes exhibited high sedentary lifestyle and low engagement in health-related physical activity. Taken together, the finding underscore the importance of physical activity in the management of type 2 diabetes and the overall health of persons with type 2 diabetes, and provides a platform for further research into tailored physical activity interventions incorporating walking for this population. Such physical activity interventions would help better manage type 2 diabetes, improve the quality of life of persons with type 2 diabetes and consequently reduce the burden of type 2 diabetes.

## Supporting information

S1 File (DOCX)

## References

[1] Zimmet PZ, McCarty DJ, De Courten MP. The global epidemiology of non-insulin-dependent diabetes mellitus and the metabolic syndrome. Journal of Diabetes and its Complications. 1997 Mar 1;11(2):60–8. doi: 10.1016/s1056-8727(96)00090-6 9101389

[2] Asamoah-Boaheng M, Sarfo-Kantanka O, Tuffour AB, Eghan B, Mbanya JC. Prevalence and risk factors for diabetes mellitus among adults in Ghana: a systematic review and meta-analysis. International health. 2019;11(2):83–92. doi: 10.1093/inthealth/ihy067 30285118

[3] Sarah D. Prevalence of diabetes mellitus and resources available for its management in the Cape Coast Metropolis. ISABB Journal of Health and Environmental Sciences. 2011;1(1):1–7.

[4] Ministry of Health Ghana (2012). National policy for the prevention and control of chronic non-communicable diseases in Ghana. Available at URL: https://www.iccp-portal.org/sites/default/files/plans/national_policy_for_the_prevention_and_control_of_chronic_non-communicable_diseases_in_ghana(1).pdf. 2012. Accessed on 5/5/2021.

[5] Amoah AG, Owusu SK, Adjei S. Diabetes in Ghana: a community based prevalence study in Greater Accra. Diabetes research and clinical practice. 2002;56(3):197–205. doi: 10.1016/s0168-8227(01)00374-6 11947967

[6] Mbanya JC, Motala AA, Sobngwi E, Assah FK, Enoru ST. Diabetes in sub-saharan africa. The lancet. 2010 Jun 26;375(9733):2254–66. doi: 10.1016/S0140-6736(10)60550-8 20609971

[7] Godman B, Basu D, Pillay Y, Mwita JC, Rwegerera GM, Anand Paramadhas BD, et al. Review of ongoing activities and challenges to improve the care of patients with type 2 diabetes across Africa and the implications for the future. Frontiers in pharmacology. 2020;11:108. doi: 10.3389/fphar.2020.00108 32265688 PMC7098994

[8] Khan MA, Hashim MJ, King JK, Govender RD, Mustafa H, Al Kaabi J. Epidemiology of type 2 diabetes–global burden of disease and forecasted trends. Journal of epidemiology and global health. 2020;10(1):107. doi: 10.2991/jegh.k.191028.001 32175717 PMC7310804

[9] Atun R, Davies JI, Gale EA, Bärnighausen T, Beran D, Kengne AP, et al. Diabetes in sub-Saharan Africa: from clinical care to health policy. The lancet Diabetes & endocrinology. 2017;5(8):622–67. doi: 10.1016/S2213-8587(17)30181-X 28688818

[10] Awadalla AW, Ohaeri JU, Tawfiq AM, Al-Awadi SA. Subjective quality of life of outpatients with diabetes: comparison with family caregivers’ impressions and control group. Journal of the National Medical Association. 2006;98(5):737. 16749649 PMC2569271

[11] World Health Organization. Global status report on noncommunicable diseases 2014. Geneva, Switzerland: World Health organization, 2014. Google Scholar. 2012.

[12] Colberg SR, Sigal RJ, Yardley JE, Riddell MC, Dunstan DW, Dempsey PC, et al. Physical activity/exercise and diabetes: a position statement of the American Diabetes Association. Diabetes care. 2016;39(11):2065–79. doi: 10.2337/dc16-1728 27926890 PMC6908414

[13] Sigal RJ, Kenny GP, Wasserman DH, Castaneda-Sceppa C. Physical activity/exercise and type 2 diabetes. Diabetes care. 2004;27(10):2518–39. doi: 10.2337/diacare.27.10.2518 15451933

[14] Balk EM, Earley A, Raman G, Avendano EA, Pittas AG, Remington PL. Combined diet and physical activity promotion programs to prevent type 2 diabetes among persons at increased risk: a systematic review for the Community Preventive Services Task Force. Annals of internal medicine. 2015;163(6):437–51. doi: 10.7326/M15-0452 26167912 PMC4692590

[15] Çolak TK, Acar G, Dereli EE, Özgül B, Demirbüken İ, Alkaç Ç, et al. Association between the physical activity level and the quality of life of patients with type 2 diabetes mellitus. Journal of physical therapy science. 2015;28(1):142–7. 10.1589/jpts.28.142 PMC4755992 26957746

[16] Ekelund U., Ward H.A., Norat T., Luan J.A., May A.M., Weiderpass E., et al. Physical activity and all-cause mortality across levels of overall and abdominal adiposity in European men and women: the European Prospective Investigation into Cancer and Nutrition Study (EPIC). The American journal of clinical nutrition. 2015;101(3):613–621. doi: 10.3945/ajcn.114.100065 25733647 PMC4340064

[17] Fernandez-Navarro P, Aragones MT, Ley V. Leisure-time physical activity and prevalence of non-communicable pathologies and prescription medication in Spain. PLoS One. 2018;13(1):e0191542. doi: 10.1371/journal.pone.0191542 29352280 PMC5774808

[18] Marwick TH, Hordern MD, Miller T, Chyun DA, Bertoni AG, Blumenthal RS, et al. Exercise training for type 2 diabetes mellitus: impact on cardiovascular risk: a scientific statement from the American Heart Association. Circulation. 2009;119(25):3244–62. doi: 10.1161/CIRCULATIONAHA.109.192521 19506108

[19] Schellenberg ES, Dryden DM, Vandermeer B, Ha C, Korownyk C. Lifestyle interventions for patients with and at risk for type 2 diabetes: a systematic review and meta-analysis. Annals of internal medicine. 2013;159(8):543–51. doi: 10.7326/0003-4819-159-8-201310150-00007 24126648

[20] Craig CL, Marshall AL, Sjöström M, Bauman AE, Booth ML, Ainsworth BE, et al. International physical activity questionnaire: 12-country reliability and validity. Med Sci Sports Exerc, 2003, 35: 1381–1395. doi: 10.1249/01.MSS.0000078924.61453.FB 12900694

[21] Boyer JG, Earp JA. The development of an instrument for assessing the quality of life of people with diabetes: Diabetes-39. Medical care. 1997;1:440–53. 10.1097/00005650-199705000-00003 9140334

[22] Abonie US, Hettinga FJ. Effect of a tailored activity pacing intervention on fatigue and physical activity behaviours in adults with multiple sclerosis. International Journal of Environmental Research and Public Health. 2021;18(1):17. 10.3390/ijerph18010017 PMC7792946 33375123

[23] Osei-Yeboah J, Owiredu W, Norgbe G, Obirikorang C, Lokpo S, Ashigbi E, et al. Physical activity pattern and its association with glycaemic and blood pressure control among people living with diabetes (PLWD) in the Ho municipality, Ghana. Ethiopian journal of health sciences. 2019;29(1). 10.4314/ejhs.v29i1.3 PMC6341429 30700949

[24] SPSS I. IBM SPSS statistics for windows. Armonk, New York, USA: IBM SPSS. 2013;2.

[25] Daniele TM, Bruin VM, Oliveira DS, Pompeu CM, Forti AC. Associations among physical activity, comorbidities, depressive symptoms and health-related quality of life in type 2 diabetes. Arquivos Brasileiros de Endocrinologia & Metabologia. 2013;57:44–50. doi: 10.1590/s0004-27302013000100006 23440098

[26] IPAQ Research Committee. Guidelines for data processing and analysis of the International Physical Activity Questionnaire (IPAQ)-short and long forms. http://www.ipaq.ki.se/scoring.pdf. 2005.

[27] Henson J, Yates T, Biddle SJ, Edwardson CL, Khunti K, Wilmot EG, et al. Associations of objectively measured sedentary behaviour and physical activity with markers of cardiometabolic health. Diabetologia. 2013;56(5):1012–20. doi: 10.1007/s00125-013-2845-9 23456209

[28] Banson AN, Boateng BA, Abonie US, Mensah YA, Yarfi CA, Kofi-Bediako Woyram, et al. Knowledge of physical activity, physical activity level and waist-to-hip ratio in adults with diabetes in a Ghanaian municipality. Ghana Med J 2023; 57(2):112–21. 10.4314/gmj.v57i2.5 PMC10846653 38504761

[29] Rizka M, Ambardini RL, Yudhistira D. The effect of walking exercise on blood pressure and blood glucose in the elderly. Int. J. Kinesiol. Sports Sci. International Journal of Kinesiology and Sports Science. 2022;10(1):30–5.

[30] Abonie US, Hoekstra F, Seves BL, Woude LHVvd, Dekker R, Hettinga FJ. Associations between Activity Pacing, Fatigue, and Physical Activity in Adults with Multiple Sclerosis: A Cross Sectional Study. Journal of Functional Morphology and Kinesiology. 2020; 5(2):43. doi: 10.3390/jfmk5020043 33467259 PMC7739300

